# Clinical Holistic Medicine: Pilot Study on the Effect of Vaginal Acupressure (Hippocratic Pelvic Massage)

**DOI:** 10.1100/tsw.2006.340

**Published:** 2006-04-14

**Authors:** Søren Ventegodt, Birgitte Clausen, Joav Merrick

**Affiliations:** ^1^ The Quality of Life Research Center, Teglgårdstræde 4-8, Copenhagen K, Denmark; ^2^ Research Clinic for Holistic Medicine, Copenhagen, Denmark; ^3^ Nordic School of Holistic Medicine, Copenhagen, Denmark; ^4^ The Scandinavian Foundation for Holistic Medicine, Sandvika, Norway; ^5^ Vejlby Lokalcenter, Vejlby, Denmark; ^6^ National Institute of Child Health and Human Development, Beer-Sheva, Israel; ^7^ Center for Multidisciplinary Research in Aging, Faculty of Health Sciences, Ben Gurion University, Beer-Sheva, Israel; ^8^ Office of the Medical Director, Division for Mental Retardation, Ministry of Social Affairs, Jerusalem, Israel

**Keywords:** quality of life, QOL, human development, holistic medicine, holistic health, repressed memory, body memory, post-traumatic stress, sexuality, ethics, Denmark

## Abstract

This is a pilot study of 20 female patients with a long history of sexual problems (mean is 8.92 years) who received vaginal acupressure (VA) with a quantitative and qualitative evaluation: 56% experienced help and none reported setbacks, 89% rated the treatment to be of high quality, and 89% rated it as valuable. After the treatment, most reported their problems to be less serious and their general quality of life improved. Only 17% reported minor or temporary side effects. VA was found statistically and clinically significant (*p* < 0.05, improvement more than 0.5 step on a 5-point Likert scale) to help patients with chronic genital pains, pain or discomfort during sexual intercourse, lack of desire or orgasm, and subjective sexual insufficiency, and all patients taken as one group (about 1 step up a 5-point Likert scale). Self-evaluated physical and mental health was significantly improved for the total group; the relationship with partner, the subjective sexual ability, and the quality of life that were measured with QOL1 and QOL5 questionnaires were all significantly improved. VA or Hippocratic pelvic massage is technically a simple procedure corresponding to the explorative phase of the standard pelvic examination, supplemented with the patient’s report on the feelings provoked followed by processing and integration of these feelings, but ethical aspects are complicated. Acupressure through the vagina/pelvic massage must be done according to the highest ethical standard with great care, after obtaining consent and the necessary trust of the patient within the framework of the local laws. It must be followed by conversational therapy and further holistic existential processing.

## INTRODUCTION

Hippocrates (460–377 BCE), the "father of medicine", and the physicians of his time were aware of female sexual disease and treatments included different physical procedures focused on the female pelvis, like smoking the vagina and massaging the pelvis[1]. For various reasons, these treatments were later abandoned and some authors even found it a form of abuse by a medical profession with insufficient ethics[2]. In holistic medicine, the physician and his patients are almost always very "close" and ethics is a subject of utmost importance. The practice of pelvic massage might even have been the cause and the need for medical ethics and the ability to practice this procedure might be the very reason why Hippocrates invented his strict medical ethics in the first place[3].

The technique of vaginal acupressure (VA) has been reviewed, developed, and tested with a number of patients at the Research Clinic for Holistic Medicine in Copenhagen[3]. The purpose of the present study was to evaluate the procedure on a larger number of patients and investigate the effects of their quality of life.

## ACUPRESSURE THROUGH THE VAGINA

Many women have problems related to their pelvis and its organs, dominated by sufferings of the sexual organs, problems of the urinary tract, the locomotor system, and the intestines[4]. Another large group of patients have "nonanatomic" pelvic pains and discomforts of presumably psychosomatic nature, very difficult to treat with biomedicine, but seem to react better to psychosomatic treatments[5,6]. From a holistic medical perspective, the problems are often caused by unsolved emotional problems that have been repressed into the pelvis and its organs (repressed memory or body memory). The emotional problems are related to negative beliefs about self, gender, body, organs, and sexuality.

In this pilot study, we have included 20 women treated with acupressure through the vagina at the Research Clinic for Holistic Medicine in Copenhagen. All patients presented with some problem(s) related to female sexuality. The study tested the hypothesis that holistic sexology with this procedure can heal old wounds on the body and soul in order to improve sexual ability, satisfaction, and quality of life in general. As in all other holistic therapy, the healing process has three obligatory steps that we sum with the words: feel, understand, and let go[7,8,9]. First, the emotions have to be felt again. We call this phase "putting feelings onto the body". Then the patient has to find words, verbalize the emotions, and understand where the problems are coming from. We call this "putting words on the feelings". Finally, the person healing has to let go of the negative attitudes and decisions that were made when the trauma was caused. We call this "putting consciousness in the words". In our clinical work, we use the therapeutic staircase, which gives us the best insurance that we do not use a more invasive and potentially dangerous technique than necessary[10], or as Hippocrates said: "Declare the past, diagnose the present, foretell the future; practice these acts. As to diseases, make a habit of two things — to help, or at least to do no harm." The Greek "First, do no harm" became "Primum non nocere" in Latin, a translation of the original perhaps, but some sources attribute "Primum non nocere" to the Roman physician Claudius Galenus of Pergamum (131–201 AD), better known in English as Galen. The procedure of acupressure through the vagina always builds on earlier sessions of acceptance through touch, which again come after sessions of emotional healing, trust, and holding, and always beginning with "love and care" for the patient.

This knowledge of healing life — improving health, quality of life, and ability in one integrated movement — is well known and described in a number of books from the cradle of medical sciences on the island of Cos around 300 BCE, known as Corpus Hippocraticum. Hippocrates was held to be the best physician of his time and father of the first scientific system of holistic healing. It is interesting that massaging the pelvis through its openings was an acknowledged method in ancient Greece[1] and was in normal use throughout Europe for centuries[2]. This necessitated the very stringent medical ethics that were founded precisely by Hippocrates, probably with the purpose that he himself and his many pupils could give this kind of treatment as mentioned above. Massage of the pelvic structures of a woman through the vagina and anus could, among other things, heal disturbances in the female energy system known as a disease called "hysteria" (from the Greek word for uterus, *hystera*). The treatment was in use in most of the western world until the industrial revolution, when it was condemned as pornographic and no longer an acceptable medical treatment.

Today, after the sexual revolution in the 1960s and 1970s, some therapists again work through the vagina and anus with this kind of therapy, either by using their hand to cure sexual and other problems[10], or by using a vibrant penis substitute (a "dildo") to cure incontinence[11] or orgasmic problems[12]. The Danish physiotherapist Birgitte Bonde reports that one to six sessions with the vibrator can help many incontinent women who are not sufficiently helped by the standard program of training the pelvic floor[11]. The rationale for the use of the vibrator is that the women cannot get in contact with their own pelvis, as they "cannot find their pelvic floor", presumably because they have completely eradicated some of the pelvic structures from their inner description of their own bodies.

It is important to understand or realize that the procedure of acupressure through the vagina is the same exploratory part of the standard pelvic examination by the physician or gynecologist, but with acupressure done so slowly that the woman can feel the emotions held by the different tissues contacted by the finger of the physician[13].

## METHODS

Twenty female patients received VA treatment for different sexual problems: chronic pain in the genitals (vulvodynia), pain or discomfort during sexual intercourse, problems with sexual desire, orgasmic malfunctioning, and other sexual inadequacy often combined with low self-esteem and mental problems related to gender and sexuality.

It is important to notice that we introduced a slow pelvic examination with a therapeutic element, relevant for a wide range of psychosomatic disturbances related to gender and sexuality, from infertility to gynecological and sexual psychosomatic problems and the long-term consequences of childhood sexual abuse[13]. On one hand, for a clinical practice, this opens up many beneficial and healing opportunities for the patient, because it allows a much closer and more intimate relationship between the patient and the physician that has been the traditional practice, but on the other hand, this procedure also has several disadvantages. In many cultures, it cannot be practiced due to cultural or religious reasons, and since the sexual taboo is so strong, the female may experience the process as overwhelming or even insulting. In the U.S., it might be practically impossible to follow our recommendation in many cases because of the time consumption, economics, and reimbursement issues of this culture and the heavy "malpractice culture" in that country.

The most difficult problem of this procedure seems to be that it makes it very difficult to be sure that the procedure and all the involved steps are always necessary and rational. This procedure and the cultural issues involved means that it has a high potential for malpractice, but this can be minimized by the following steps: (1) before the procedure is done, the patient must read about it with at least one case study to illustrate, to fully understand the emotional and existential implications of the procedure, so she has time to contemplate and make her decision of whether to accept or not; (2) the procedure is also orally presented by the physician to the patient before she signs the contract; (3) the physician must be in supervision to discuss the problems, if any, about borders, intimacy, emotional and sexual issues. (Close supervision and full intercollegial openness is the best prevention of malpractice, as malpractice often occurs with physicians without a network and without openness about what is going on in their clinic[13]); and (4) a third person must be present at the examination.

The participants completed the QOL5 and QOL1 questionnaires[14] before and after treatment. After the treatment, they were interviewed about the side effects of the treatment, their experience of the treatment, and the experienced quality and value of the treatment (see the questionnaire for the semistructured interview in the Appendix). The statistical method for estimating the level of significance was a paired t-test in the SPSS statistical program. Informed consent was given before the procedure and the interview. The procedure was performed by a male physician and a female nurse present, except in a few cases where this was not possible.

## RESULTS

Of the 20 patients, 18 participated in the study. Of these, 6 suffered from genital chronic pain or discomfort, 15 suffered from problems with sexual desire or orgasmic malfunctioning, and 17 also had other sexual inadequacies (see Table 1).

**TABLE 1**
**The Therapeutic Effect of VA Using QOL5+QOL1 Before and After the Treatment**

| Group* | Question** | | | | | | Delta (7-6) Mean (*p*-Value) | Question** | | | | |
|---|---|---|---|---|---|---|---|---|---|---|---|---|
| | 2 Mean (Years) | 3 Mean (Weeks with One VA Treatment Every 2nd Week) | 4 Mean | 5 Mean | 6 Mean | 7 Mean | | 11 Mean | 12 Mean | 13 Mean | 14 Mean | 15 Mean |
| 1 (n = 2) | 3.00 | 3.00 | 1.50 | 1.00 | 4.50 | 4.50 | 0.00 (0.50) | 1.00 | 2.50 | 2.50 | 2.00 | 3.00 |
| 2 (n = 6) | 11.17 | 2.83 | 1.67 | 1.50 | 4.00 | 3.00 | –1.00 (0.09) | 1.17 | 1.67 | 2.67 | 1.50 | 1.83 |
| 3 (n = 12) | 7.38 | 9.25 | 1.83 | 1.67 | 3.83 | 3.17 | –0.66 (0.136) | 1.00 | 1.58 | 2.58 | 1.67 | 1.75 |
| 4 (n = 12) | 7.96 | 9.50 | 1.67 | 1.59 | 3.83 | 3.17 | –0.66 (0.071) | 1.00 | 1.58 | 2.58 | 1.59 | 1.75 |
| 5 (n = 17) | 9.15 | 8.53 | 1.82 | 1.65 | 3.81 | 3.00 | –0.81 (0.022) | 1.06 | 1.53 | 2.59 | 1.59 | 1.65 |
| 6 (n = 5) | 9.40 | 13.80 | 1.40 | 1.60 | 3.60 | 2.20 | –1.40 (0.005) | 1.00 | 1.20 | 2.40 | 1.40 | 1.80 |
| 7 (n = 4) | 13.25 | 5.25 | 1.50 | 1.50 | 4.25 | 3.00 | –1.25 (0.80) | 1.00 | 1.75 | 2.75 | 2.00 | 2.00 |
| ALL (n = 18) | 8.92 | 8.11 | 1.78 | 1.61 | 3.82 | 3.06 | –0.76 (0.023) | 1.06 | 1.50 | 2.61 | 1.56 | 1.61 |
| Group A (1+2) (n = 6) | 11.17 | 2.83 | 1.67 | 1.50 | 4.00 | 3.00 | –1.00 (0.089) | 1.67 | 1.67 | 2.67 | 1.50 | 1.83 |
| Group B (3+4) (n = 15) | 6.97 | 8.87 | 1.73 | 1.60 | 3.80 | 3.13 | –0.67 (0.067) | 1.00 | 1.53 | 2.52 | 1.60 | 1.67 |
| Group C (5+6) (n = 17) | 9.15 | 8.53 | 1.82 | 1.65 | 3.81 | 3.00 | –0.81 (0.022) | 1.06 | 1.53 | 2.59 | 1.59 | 1.65 |

* 1, Chronic pain; 2, pain/discomfort during sexual intercourse; 3, problems with sexual desire; 4, orgasmic dysfunction; 5, self-confidence/psychological problems related to gender and sexuality; 6, other subjects concerning sexual inadequacy; 7, other subjects; Group A, pain or discomfort; Group B, lack of desire or orgasm; Group C, subjective sexual inadequacy.

** Question numbers correspond to the questions and their rating scales on quality, value, and efficiency of the treatment found in the Appendix.

Ten patients felt that they were helped by the VA treatment, while six patients did not feel any change in their symptoms. None of the patients felt any setbacks. Success rate was 56%.

The duration of the treatment was an average of 8 weeks and 4 sessions, once every fortnight. Most patients rated their problem as serious before the treatment, and neither serious nor unserious after the treatment; the average improvement here was 1 step up a 5-point Likert scale, independent of suffering. This is a positive and clinically significant improvement, as experienced by the patients. Most patients would choose the treatment again if they needed it. Most patients had good expectations for their future sex-life after the treatment. Most patients rated the quality of the treatment to be very high (10 cases), or high (6), and two rated it as average. Most patients rated the value of the treatment to be very high (13) or high (3), while one rated the value low and one as very low. Thus, 89% of the patients found the treatment of high quality and 89% of the patients found it valuable. Most patients had an understanding and supporting reaction from their surroundings (family, friends, and partner), if they shared the information about receiving the treatment.

In the beginning of treatment, the patients presented a number of different symptoms categorized into the following seven subgroups:

1. Chronic genital pain
2. Pain/discomfort during sexual intercourse
3. Problems with sexual desire
4. Orgasmic malfunctioning
5. Self-esteem/mental problems related to gender and sexuality
6. Other gender or sexual inadequacy
7. Other problems

Because of the limited number of respondents, the patients were also grouped according to their problems in general: Group A (1+2) was the patients who suffered from chronic genital pains or discomforts, group B (3+4) was the patients who suffered from lack of desire or orgasm, while group C (5+6) suffered from other forms of sexual problems.

The following symptom groups were found to be helped statistically and were clinically significant ($p < 0.05$, improvement more than 0.5 step on a 5-point Likert scale to the question “How serious was the problem before and after?”):

- Group (2) “Pain/discomfort during sexual intercourse”
- Group A (1+2) “Genital pain or discomfort”
- Group B (3+4) “Lack of desire or orgasm”
- Group C (5+6) “Subjective sexual insufficiency”
- All the patients taken as one group

The treatment of all groups A–C had a good and remarkable effect on the specific problems (about 1 step up the Likert scale). Self-evaluated physical and mental health was also significantly and clinically improved for the total group of patients; also the relationship with partner, the subjective sexual ability, and the quality of life measured with QOL1 and QOL5 questionnaires (see Tables 1–3). It is important to notice the very large improvements in most of the dimensions.

## Side Effects

Of the 18 patients, only 3 (17%) reported side effects:

1. Feelings of shame and guilt, a tear caused by an old scar being worked on, 4–5 days of bleeding, and a little tenderness in an area known to the patient as a sore spot since childhood.
2. Genital soreness and a disturbed feeling in the body for 14 days.

3. "For some time I felt the pain I normally feel during intercourse: a soreness and an intense feeling of shame, and a strange feeling of weakness in the pelvic floor."

All side effects were temporary and none could be considered harmful. Acupressure through the vagina, therefore, seems to be practically without side effects or is at least at the same level as the standard normal gynecological examination.

**TABLE 2A.**
**Clinical Effect of Vaginal Acupressure. Subjective physical and mental health, quality of relationship with partner before and after VA treatment.**

| Groups/Question | QOL5-1$_{before}$ Subjective physical health<br>Very good (1) to very bad (5)<br>Mean | QOL5-1$_{after}$ Subjective physical health<br>Very good (1) to very bad (5)<br>Mean | Delta QOL5-1<br>Mean (P-value) | QOL5-2$_{before}$ Subjective mental health<br>Very good (1) to very bad (5)<br>Mean | QOL5-2$_{after}$ Subjective mental health<br>Very good (1) to very bad (5)<br>Mean | Delta QOL5-2 Mean (P-value) | QOL5-5$_{before}$ Relationship with partner<br>Very good (1) to very bad (5)<br>Mean | QOL5-5$_{after}$ Relationship with partner<br>Very good (1) to very bad (5)<br>Mean | Delta QOL5-5 Mean (P-value) |
|---|---|---|---|---|---|---|---|---|---|
| **1 (N=2)** | 5.00 | 4.50 | **-0.50 (0.50)** | 3.00 (0.00) | 3.50 (0.71) | **-0.50 (0.50)** | 3.50 (0.00) | 3.42 (0.12) | **-0.08 (0.50)** |
| **2 (N=6)** | 3.17 | 2.50 | **-0.67 (0.102)** | 3.50 | 2.83 | **-0.67 (0.328)** | 3.67 | 2.97 | **-0.70 (0.058)** |
| **3 (N=12)** | 3.00 | 2.41 | **-0.58 (0.012)** | 3.17 | 2.58 | **-0.58 (0.152)** | 3.47 | 2.71 | **-0.76 (0.011)** |
| **4 (N=12)** | 3.17 | 2.41 | **-0.75 (0.002)** | 3.17 | 2.75 | **-0.42 (0.241)** | 3.42 | 2.94 | **-0.47 (0.071)** |
| **5 (N=17)** | 2.71 | 2.29 | **-0.41 (0.069)** | 3.35 | 2.47 | **-0.88 (0.011)** | 3.44 | 2.68 | **-0.76 (0.002)** |
| **6 (N=5)** | 2.60 | 2.00 | **-0.60 (0.070)** | 3.00 | 2.60 | **-0.40 (0.477)** | 3.23 | 2.90 | **-0.33 (0.408)** |
| **7 (N=4)** | 3.00 | 2.75 | **-0.25 (0.391)** | 3.00 | 2.50 | **-0.50 (0.495)** | 3.13 | 2.42 | **-0.71 (0.276)** |
| **ALL (N=18)** | 2.83 | 2.38 | **-0.44 (0.042)** | 3.33 | 2.50 | **-0.83 (0.012)** | 3.44 | 2.72 | **-0.72 (0.003)** |
| **GroupA (1+2) (N=6)** | 3.17 | 2.50 | **-0.67 (0.102)** | 3.50 | 2.83 | **-0.67 (0.328)** | 3.67 | 2.97 | **-0.70 (0.058)** |
| **GroupB (3+4) (N=15)** | 3.07 | 2.40 | **-0.67 (0.001)** | 3.20 | 2.60 | **-0.60 (0.082)** | 3.41 | 2.81 | **-0.60 (0.021)** |
| **GroupC (5+6) (N=17)** | 2.71 | 2.29 | **-0.41 (0.069)** | 3.35 | 2.47 | **-0.88 (0.011)** | 3.44 | 2.68 | **-0.76 (0.002)** |

* 1, Chronic pain; 2, pain/discomfort during sexual intercourse; 3, problems with sexual desire; 4, orgasmic dysfunction; 5, self-confidence/psychological problems related to gender and sexuality; 6, other subjects concerning sexual inadequacy; 7, other subjects; Group A, pain or discomfort; Group B, lack of desire or orgasm; Group C, subjective sexual inadequacy.

** Question numbers correspond to the questions and their rating scales on quality, value, and efficiency of the treatment found in the Appendix.

**TABLE 2B.**
**CLINICAL EFFECT OF VAGINAL ACUPRESSURE.**
**Subjective sexual ability, and QOL (quality of life) before and after VA treatment.**

| **Groups/Question** | **$A_{before}$ Subjective sexual ability** Very good (1) to very bad (5) **Mean** | **$A_{after}$ Subjective sexual ability** Very good (1) to very bad (5) **Mean** | **Delta A (before, after) Mean (P-value)** | **QOL $5_{before}$ Quality of life** Very good (1) to very bad (5) **Mean** | **$QOL5_{after}$ Quality of life** Very good (1) to very bad (5) **Mean** | **Delta QOL5 Mean (P-value)** |
|---|---|---|---|---|---|---|
| **1 (N=2)** | 5.00 | 4.00 | **-1.00 (0.50)** | 5.00 | 4.50 | **-0.50 (0.50)** |
| **2 (N=6)** | 4.17 | 3.17 | **-1.00 (0.041)** | 4.33 | 3.00 | **-1.33 (0.010)** |
| **3 (N=12)** | 4.08 | 3.00 | **-1.08 (0.012)** | 3.70 | 2.83 | **-0.87 (0.064)** |
| **4 (N=12)** | 4.00 | 3.25 | **-0.75 (0.082)** | 3.92 | 3.17 | **-0.75 (0.069)** |
| **5 (N=17)** | 3.88 | 3.00 | **-0.88 (0.014)** | 3.76 | 2.76 | **-1.00 (0.005)** |
| **6 (N=5)** | 3.40 | 3.20 | **-0.20 (0.799)** | 3.80 | 3.00 | **-0.80 (0.099)** |
| **7 (N=4)** | 3.50 | 2.25 | **-1.25 (0.080)** | 3.25 | 3.00 | **-0.25 (0.789)** |
| **ALL (N=18)** | 3.94 | 3.00 | **-0.94 (0.007)** | 3.83 | 2.83 | **-1.00 (0.003)** |
| **GroupA (1+2) (N=6)** | 4.17 | 3.17 | **-1.00 (0.041)** | 4.33 | 3.00 | **-1.33 (0.010)** |
| **GroupB (3+4) (N=15)** | 4.00 | 3.07 | **-0.93 (0.021)** | 3.80 | 2.93 | **-0.87 (0.022)** |
| **GroupC (5+6) (N=17)** | 3.88 | 3.00 | **-0.88 (0.014)** | 3.76 | 2.76 | **-1 (0.011)** |

* 1, Chronic pain; 2, pain/discomfort during sexual intercourse; 3, problems with sexual desire; 4, orgasmic dysfunction; 5, self-confidence/psychological problems related to gender and sexuality; 6, other subjects concerning sexual inadequacy; 7, other subjects; Group A, pain or discomfort; Group B, lack of desire or orgasm; Group C, subjective sexual inadequacy.

** Question numbers correspond to the questions and their rating scales on quality, value, and efficiency of the treatment found in the Appendix.

**TABLE 3**
**The Clinical Effect of VA (Paired Sample t-Test for the Whole Group, n = 18)**

| | | Mean Difference | SD | t | df | Sig. |
|---|---|---|---|---|---|---|
| Pair 1 | Self-evaluated physical health; QOL5–1 (before, after) | 0.44 | 0.86 | 2.20 | 17 | 0.042 |
| Pair 2 | Self-evaluated mental health; QOL5–2 (before, after) | 0.83 | 1.25 | 2.83 | 17 | 0.012 |
| Pair 3 | Self–assessed sexual ability; A (before, after) | 1.00 | 1.24 | 3.43 | 17 | 0.003 |
| Pair 4 | QOL 1 (before, after) | 0.72 | 0.87 | 3.53 | 17 | 0.003 |
| Pair 5 | QOL 5 (before, after) | 0.94 | 1.30 | 3.07 | 17 | 0.007 |
| Pair 6 | Improvement related to original problem; Q6,Q7 (before, after) | 0.76 | 1.25 | 2.52 | 17 | 0.023 |

## Qualitative Evaluation of the Effect of the VA Treatment

Below we present the experiences of the treatment and its results from nine patients that gave detailed information on question 10 in the questionnaire (see Appendix).

**Female, aged 23 years, with psychological problems related to gender, sexuality, and orgasmic dysfunction**

The patient experienced that suppressed and unconscious material surfacing during the sessions. In therapy she got feelings like “everything is wrong”, and at the same time she saw and felt how her problem changed and brought her further towards her personal development; she fluctuated between joy and “darkness” in the sessions.

**Female, aged 27 years, with psychological problems related to gender, sexuality, orgasmic dysfunction, and lack of sexual desire**

In the beginning of the therapy the patient found it very difficult to be touched on her body. Gradually as she progressed that became much easier. “I had problems letting him [the therapist] through my façade and let him touch me. I expected and hoped for a miracle to occur without my participation, since that would be far too embarrassing. I expressed my will to solve my problems by showing up in therapy, but I never took an active part in the process. I did not have the courage”.

**Female, aged 22 years, with psychological problems related to gender, sexuality, orgasmic dysfunction, and lack of sexual desire**

The patient felt safe with the therapy and able to let out all the feelings surfacing during the sessions. She experienced different states of her development, like at first being very small, and later the wish to be sexual and feel desire. She went through a lot of inner resistance. Later she realized that she was full of hate and disgust, which stopped her from giving in to her vulnerability and open up to her sexuality. “I was so full of hate. My pelvis was shaking, but it felt so good and relieving. Afterwards I felt incredibly wonderful.” The patient experienced that she, due to the acceptance of her by the physician (SV), managed to accept her own gender. She went through a lot of shame, sadness, anger and feelings of forced penetration and got all the way through to a point, where she could enjoy and express her sexual desire and achieve fantastic orgasms with her partner.

**Female, aged 23 years, with orgasmic dysfunction and lack of sexual desire**

The patient experienced the treatment as very painful emotionally, as a result of psychological traumas caused by sexual abuse in her childhood. She also described the relief in confronting the old pain with the support, acceptance and "love" or care of her therapist and holders. She processed a lot of shame and guilt. "I now have a more natural and accepting relation to my own sexuality. I no longer have the tensions connected to having sex with a boyfriend. I now feel the desire to have sex and I do not want to hold myself back the way I used to. Now I am able to accept male sexuality, which used to be a big problem for me. I realize that you get ill, if you do not accept your own nature."

**Female, aged 29 years, with huge discomfort when touched directly on her genitals (primary vulvodynia)**

VA was a highly painful procedure for the patient, who at first thought of her therapist as being evil. A nurse was present at the sessions to support the patient in processing her old pains of humiliation and embarrassment with her own sexuality – and as she slowly got through the old traumas during this therapy, the "genitals changed into a natural appearance. I have accepted my sexuality. It feels good to have sex with my boyfriend and the pain is gone. My labia changed shape – like withdrawing into my body like it accepted them as a part of me. The psychological and the physical part of me blended together and now I feel like a whole person."

**Female, aged 22 years, virgin with chonic pain (primary vulvodynia)**

In the beginning of the therapy she thought of the treatment as barrier breaking. But as she also felt great trust in the methods and in her therapist she was able to give herself in to the process. She felt how she immediately began to blossom, and how sexuality became a natural part of her existence. After the treatment she was able to have sex with a partner and enjoy it. About three months later new personal problems hit the surface. She felt reserved and unfeeling with the problems coming back.

**Female, aged 27 years, with orgasmic dysfunction and lack of sexual desire**

She described how VA has helped her attain great sexual liberation, the ability to feel sexual desire and to let go of all her inhibitions. She now sees herself as a sexual being. She is orgasmic functional and values sex as highly important for the quality of life.

**Female, aged 27 years, with psychological problems related to gender, sexuality and lack of sexual desire**

She hated life and hated herself. She was never able to find love anywhere. As a result of this she became numb and let herself get sexually abused by men she did not love or desire in any way. In therapy she needed to confront her old traumatic pain. At first she found it difficult to re-establish contact with her own body and had become very emotionally controlled. Letting go of this control was connected with great fear. During sessions she felt nervous and insecure, because the physician (SV) "looked at her" and she tried to escape her emotions. But as she realized that she was completely safe, she decided to let go of her control and she opened up to the experience of shame, humiliation and the huge pain in her self-abuse. She remembered how she hated herself even as a child for growing faster then her friends and being ashamed of her breasts, which made her withdraw from the world. Gradually as she went through the process and felt the acceptance from her therapist, she also began to get in touch with her self-acceptance and get a whole new experience from the treatment: "It's extremely difficult, but after a while I feel that it's really wonderful, I'm not ashamed and I just let the enjoyment spread all the way from vulva to my uterus and my whole body. It created a fantastic wild feeling, a healing energy and warmth going into my body. It felt like my heart, my breasts and my throat melted and opened up. The feeling was beyond words."

**Female, aged 30 years, with no contact to feelings and sexuality (anorgasmic)**

In therapy she worked with the condemnations that she experienced as a child regarding her sexuality. She found it extremely difficult being on the couch and was very tense. She was switching between the feeling of her boarders being trespassed and the pure trust in her therapist to realize that this treatment would be of great help. She got focused on how she suppressed her sexual desire. During the session she experienced a warm feeling of desire, but condemned it herself. She was hugely embarrassed and would not allow the sensation to be present. She got the insight that she actually did have a lot of sexual feelings, but had denied them mentally. All she had to do was to awaken them in order to get back in contact with her body. After a while the tensions disappeared. As the process proceeded she got deeper and deeper into her pain and the idea that other people would think of this as being morally reprehensible and scandalous. She started to feel great fear of condemnation and realized how she was always pre-occupied with doing "the right thing". In spite of her fear she decided to let go of this need and stop holding back: "I feel the most wonderful desire…strange, I can't explain it. I think I'm just experiencing all the desire that has been repressed during many years…It's still hard, when I realize how excited I am, but I can't fight it anymore…I hear myself breathing heavily and I feel the ecstatic sensations of orgasm waving through my body….I get the most wonderful feeling in my body and I am completely relaxed."

In conclusion, it seemed evident from the qualitative study of the patient that the majority felt helped by the VA treatment. Most patients found it valuable, even when the problem they originally presented had not disappeared. In general, the patients were satisfied with the VA treatment.

## DISCUSSION

Acupressure through the vagina must be performed according to the highest ethical standards. The holistic sexological procedures are derived from holistic existential therapy, which involves reparenting, massage and bodywork, conversational therapy, philosophical training, healing of existence during spontaneous regression to painful life events (gestalts), and close intimacy without any sexual involvement[15]. In psychology, psychiatry, and existential psychotherapy[16,17], touch is often allowed, but a sufficient distance between therapist and client must always be kept, all clothes must be kept on, and it is even recommended that the first name is not taken into use to keep the relationship as formal and correct as possible[18]. The reason for this distance is to create a safety zone that removes the danger of psychotherapy leading to sexual involvement.

The patients in holistic existential therapy and holistic sexology are often chronically sick and their situation often seems pretty hopeless, as many of them have been dysfunctional and incurable for many years or they are suffering from conditions for which there are no efficient biomedical cures. Many are also unaware of body memory or repressed memory due to earlier traumatic stress[19,20,21] and some only open up for their earlier sexual abuse through this examination, because the touch becomes the trigger between body and soul.

The primary purpose of holistic existential therapy is to improve quality of life and the secondary purpose is to improve health and ability. The severe conditions of the patients and the chronicity is what ethically justifies the much more direct, intimate, and intense methods of holistic existential therapy, which integrates many different therapeutic elements and works on many levels of the patient's existence and personality at the same time. Holistic sexology is holistic existential therapy taken into the domain of sexology. The general ethical rule is that everything that does not harm and in the end will help the patient is allowed ("first do no harm"), but we understand that this procedure is not accepted in many other countries due to sexual taboo. It is interesting, though, that this or similar techniques have been used by many physicians[22,23,24,25,26] and, in particular, alternative therapists outside the medical profession[27,28,29,30,31,32,33,34,35,36,37,38,39,40,41,42,43,44,45]. An important aspect of the therapy is that the physician must be creative and, in practice, invent a new treatment for every patient, as Yalom has suggested[46,47]. To perform the sexological technique of acupressure through the vagina, the holistic sexologist must be able to control not only his/her behavior, but also his sexual excitement to avoid any danger of the therapeutic session turning into sexual activity. The necessary level of mastery of this art can only be obtained through training, supervision, and the presence of a third person. The role of the physician is asexual and therapeutic.

In this pilot study, 20 female patients with sexual problems received acupressure through the vagina and evaluated the treatment both quantitatively and qualitatively; 56% of the patients experienced that the procedure helped them with their problem; 89% of the patients rated the treatment as of high quality, and 89% rated it as valuable. Most reported their problems to be less serious and their general quality of life improved after the treatment. Acupressure through the vagina seemed to have no serious side effects and self-evaluated physical and mental health was significantly and clinically improved for the total group of patients.

We therefore conclude that acupressure though the vagina can help many women with chronic genital pains, coital discomfort, problems with sexual desire and orgasmic malfunctioning, and other problems of female sexuality. Acupressure through the vagina thus seems to be a safe and efficient procedure, and important tool in the holistic medical toolbox. We recommend a full-scale clinical study of acupressure though the vagina. We also recommend that the patient treated with acupressure be contacted after 1–5 years to prevent and handle any potential long-term negative effects of the treatment.

## ACKNOWLEDGMENTS

This study was supported by grants from IMK Almene Fond. The quality of life research was originally approved by the Copenhagen Scientific Ethical Committee under number (KF)V.100.2123/91 and later correspondence.

## BIOSKETCHES

**Søren Ventegodt, MD,** is the director of the Nordic School of Holistic Health and Quality of Life Research Center in Copenhagen, Denmark. He is also responsible for a Clinical Research Clinic for Holistic Medicine in Copenhagen and is a popular speaker throughout Scandinavia. He has published numerous scientific or popular articles and a number of books on holistic medicine, quality of life, and quality of working life. His most important scientific contributions are the comprehensive SEQOL questionnaire, the very short QOL5 questionnaire, the integrated QOL theory, the holistic process theory, the life mission theory, and the ongoing Danish Quality of Life Research Survey, 1991–94 in cooperation with the University Hospital of Copenhagen and the late professor of pediatrics, Bengt Zachau-Christiansen, MD, PhD. E-mail: ventegodt@livskvalitet.org. Website: www.livskvalitet.org

**Birgitte Clausen, RN,** leader of a secluded ward for people with dementia. Dementia coordinator and consultant in Aarhus, Denmark with more than 10 years of experience in adult psychiatry and dementia. During the last 4 years, she has worked together with Søren Ventegodt on the “QOL as medicine project” and on developing existential holistic group therapy. Through her articles and lectures, she has disseminated “The New Culture of Dementia” in both Denmark and Japan. She has participated in the production of several videos to order to inspire relatives, medical staff, and volunteers to understand people with dementia. This project has been granted financial support from the Ministry of Social Affairs in Denmark. She has conducted the first Danish investigation on the effect of sufficient daylight on the human spirit, activity level, and biological rhythm. E-mail: villahvide@mail.dk

**Joav Merrick, MD, DMSc,** is professor of child health and human development affiliated with the Center for Multidisciplinary Research in Aging, Zusman Child Development Center, Division of Pediatrics and Community Health at the Ben Gurion University, Beer-Sheva, Israel; the medical director of the Division for Mental Retardation, Ministry of Social Affairs, Jerusalem; and the founder and director of the National Institute of Child Health and Human Development. Dr. Merrick has numerous publications in the field of child health and human development, rehabilitation, intellectual disability, disability, health, welfare, abuse, advocacy, quality of life, and prevention and received the Peter Sabroe Child Award for outstanding work on behalf of Danish Children in 1985 and the International LEGO-Prize (“The Children’s Nobel Prize”) for an extraordinary contribution towards improvement in child welfare and well being in 1987. E-mail: jmerrick@internet-zahav.net. Website: www.nichd-israel.com

## APPENDIX

Scientific examination of the effect of vaginal acupressure at the Research Clinic for Holistic Medicine, Copenhagen 2005

Patient's name ______________________________________

Date _________

Written accept of the treatment and the use of the data for scientific purpose.

I hereby accept, by my signature, to be part of the experiment of vaginal acupressure and that my data will be used for research in anonymous form. I have received the written patient formula and the article in Danish about vaginal acupressure (from Ny medicin II (New Medicine newspaper))

________________________

Signature of the patient

Responsible for data collection: Herluf Riddersholm

Responsible for treatment: Søren Ventegodt

The following questions shall be written in words or as numbers in the questionnaire below

1) What was the problem/illness?

2) How long did the patient have the problem/illness?__________________________________

3) How long did the treatment last?______________________________________________

Define the problem using the possibilities below (more answers are allowed)

1. Chronic genital pain
2. Pain/Discomfort during sexual intercourse
3. Problems with sexual desire
4. Orgasmic malfunctioning
5. Self-esteem/mental problems related to gender and sexuality
6. Other gender or sexual inadequacy
7. Other problems: _____________________

4) Was there an alternative medical treatment? 1 Yes, 2 No

5) Had the alternative treatment been tried? 1 Yes, 2 No

6) How serious was the problem to the patient before the treatment? Very serious (5) to not serious at all (1)? 5 4 3 2 1

7) How serious was the problem to the patient after the treatment? Very serious (5) to not serious at all (1)? 5 4 3 2 1

8) Describe the treatments. How was it to receive vaginal acupressure (or physical acceptance at the vulva/vagina?

________________________________________

________________________________________

9) Were there any side effects? 1 Yes, 2 No.

If yes, which occurred and for how long did they last?

________________________________________

10) Which problem/problems were solved through the treatment?

________________________________________

________________________________________

11) Would you choose this treatment again if needed? 1 Yes 2 No

12) How do you expect your sexuality to evolve in the future? (expectations to sexual capability and functioning)? Very good (1) to very bad (5). 1 2 3 4 5

13) Could your problems regarding /related to this treatment have been solved another way? Yes (1), Maybe (2) No (3). 1 2 3

14) How would you rate the quality of the treatment? Very high (1) to very low (5). 1 2 3 4 5

15) How would you rate the value of the treatment? Very high (1) to very low (5). 1 2 3 4 5

16) How was the reaction of your surroundings (family, friends, partner)?

QOL1: How would you assess the quality of your life now?

Answer: I: very high, II: high, III: neither high nor low, IV: low, V: very low

## The QOL5 Questionnaire for Clinical Databases:

Dear Mr/Mrs/Miss

In order to evaluate the benefits of appointments and treatments in the health services, we would like you to answer a few questions concerning your quality of life.

Please consider the questions carefully before answering. Then draw a circle around the most suitable answer.

QOL5-1. How do you consider your **physical health** at the moment?

1 Very good
2 Good
3 Neither good nor bad
4 Bad
5 Very bad

QOL5-2. How do you consider your **mental health** at the moment?

1 Very good
2 Good
3 Neither good nor bad
4 Bad
5 Very bad

QOL5-3. How is your relationship with your **partner** at the moment?

1 Very good
2 Good
3 Neither good nor bad
4 Bad
5 Very bad/I do not have one

QOL5-4. How are your relationships with your **friends** at the moment?

1 Very good
2 Good
3 Neither good nor bad
4 Bad
5 Very bad

QOL5-5. How do you **feel about yourself** at the moment?

1 Very good
2 Good
3 Neither good nor bad
4 Bad
5 Very bad

Please make certain that you have answered **all** the questions. Thank you for your help.

"QL5". Copyright 2001. The Quality-of-Life Research Centre, Teglgaardsstræde 4-8, DK-1452 Copenhagen.

Phone +45 33 14 11 13 Fax: +45 33 14 11 23

This questionnaire is hereby released for noncommercial, scientific use. Please apply for written permission for all commercial and/or non-scientific use to The Quality-of-Life Research Center.

QOL5 + QOL1 were applied before and after the treatment. If the QOL5 + QOL1 data before treatment were not obtained initially as intended, the patient answered it in the end twice, with both the actual and the initial values (retrospectively). In this case “retrospective” is noted in the questionnaire. Added to the QOL5+QOL1 questionnaire was also:

A) How would you rate your sexual ability these days? Very high (1) to very low (5). 1 2 3 4 5
